# Classification-based genomic prediction for early identification of high-yielding and stable soybean genotypes

**DOI:** 10.3389/fpls.2026.1770360

**Published:** 2026-04-29

**Authors:** Rafael Goncalves Marmo, Andrea Acuña, Chengjun Wu, Liliana Florez-Palacios, Derrick Harrison, Daniel Rogers, Igor Kuivjogi Fernandes, Vitor Seiti Sagae, Trenton Lee Roberts, Jeremy Ross, Qingyang Zhang, Qijian Song, Diego Jarquin, Caio Canella Vieira

**Affiliations:** 1 University of Arkansas, Division of Agriculture, Department of Crop, Soil, and Environmental Sciences, Fayetteville, AR, United States; 2 Agronomy Department, University of Florida, Gainesville, FL, United States; 3 University of Arkansas, Department of Mathematical Sciences, Fayetteville, AR, United States; 4 Soybean Genomics and Improvement Laboratory, Agricultural Research Service, United States Department of Agriculture, Beltsville, MD, United States

**Keywords:** classification-based genomic prediction, early-stage yield trials, grain yield stability, resource allocation efficiency, soybean breeding

## Abstract

Improving grain yield remains the central objective of soybean breeding programs. During early-stage yield trials, breeders often evaluate thousands of genotypes; however, limited seed availability constrains the number of tested environments and replications, reducing selection accuracy. Genomic prediction offers a promising approach to identify high-yielding and stable genotypes earlier in the breeding pipeline. The objective of this study was to develop a classification-based genomic prediction framework that directly targets advancement decisions by assigning genotypes to yield performance classes while estimating the probability of class membership to prioritize genotypes with higher confidence. A total of 1,789 soybean genotypes, ranging from maturity groups III to V, were evaluated for grain yield across 10 environments (year × location combinations) in Arkansas and Missouri during the 2023 and 2024 growing seasons. Genomic Best Linear Unbiased Predictors (GBLUPs) were obtained for each genotype in each environment, and a selection index (MSI) was calculated as the average yield deviation from the mean of the checks across the tested environments, centered at zero. This metric captures both yield and consistency across environments using a simple, check-referenced scale that is directly interpretable in breeding decisions. Genotypes were then classified as high-yielding (MSI ≥ –5), moderate (–5 > MSI ≥ –15), or low-yielding (MSI < –15). Two classification-based genomic prediction models, Generalized Linear Model via Elastic Net Regularization (GLMNet) and Random Forest (RF), were trained using the SoySNP3K BeadChip markers as predictors and the MSI-based yield classes as response categories. The MSI ranged from -32.4 to 7.2, with a small proportion of genotypes in the high-yielding class. GLMNet and RF achieved macro-averaged balanced accuracies of 0.84 and 0.83, respectively, with high specificity (0.89 for both) and sensitivity (0.78 and 0.76), and minimal extreme misclassification between low- and high-yielding classes. Compared to regression-based genomic prediction, this classification framework aligns with advancement decisions, is less sensitive to early-stage noise, and retains greater genetic diversity than GBLUP-based ranking, enabling more efficient resource allocation and more targeted advancement of promising genotypes.

## 1 Introduction

**Figure 1:**
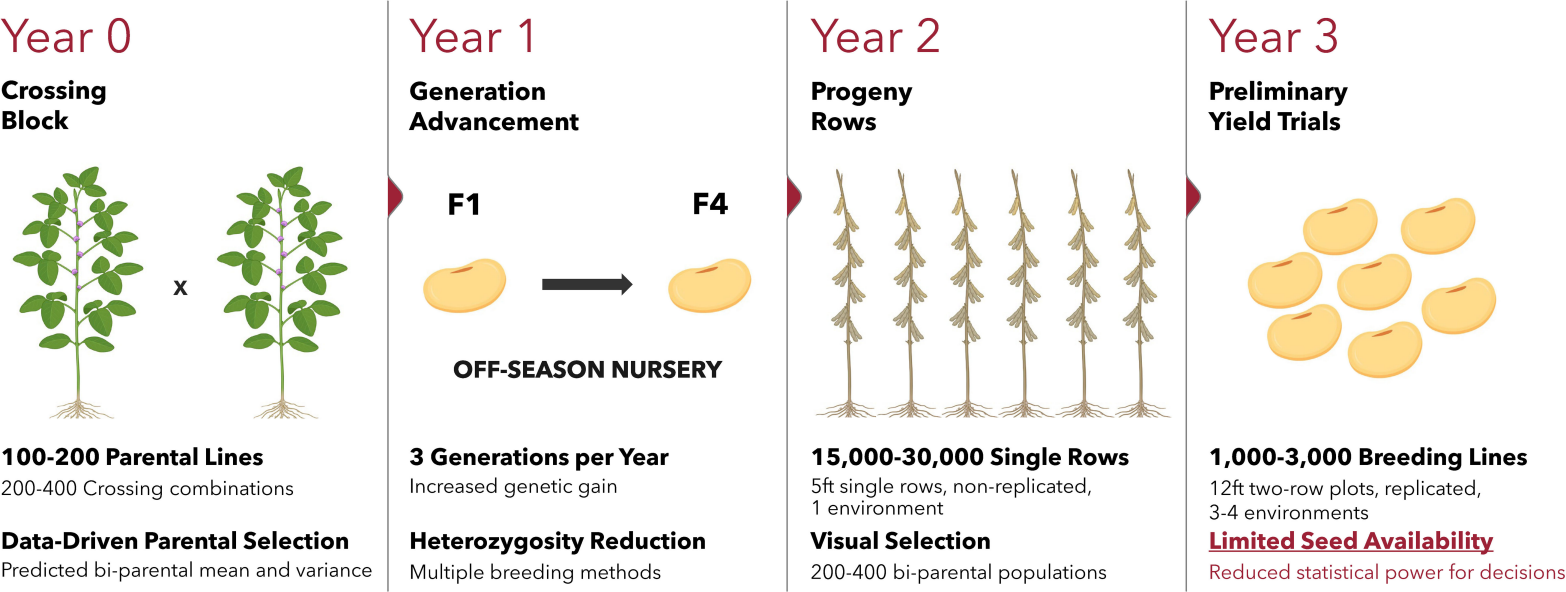
Early-stage soybean cultivar development pipeline in which the large pool of breeding lines, along with the limited seed availability, may limit testing capacity across a wide range of environments.

In the United States, a typical soybean [*Glycine max* (L.) Merr.] cultivar development pipeline progresses from early generation advancement through multi-environment yield trials to eventual germplasm or cultivar release (Vieira and Chen, 2021). During early-stage trials, breeders often evaluate thousands of genotypes, but limited seed availability restricts the number of tested environments and replications. These constraints reduce selection accuracy and intensity, particularly for quantitative traits such as grain yield that are strongly influenced by the environment (Jarquín et al., 2017) and limit the ability to assess genotype stability and adaptation across target environments (**Figure 1**; Finlay and Wilkinson, 1963). Consequently, developing genomic prediction (GP) models capable of identifying high-yielding and stable genotypes early in the breeding cycle is essential for improving resource allocation and accelerating genetic gain.

The definition of genotype stability includes both static and dynamic concepts, each with distinct implications for breeding programs. The static concept defines stability as the ability of a genotype to maintain consistent performance across environments, although such genotypes are often characterized by relatively low yield potential (Becker and Leon, 1988). In turn, the dynamic concept views stability as predictable responsiveness, where a genotype improves its performance as environmental yield potential increases (Becker and Leon, 1988). In practical breeding terms, a stable genotype is one that consistently outperforms high-yielding reference checks within a given maturity group (MG) and target environment. Developing genomic tools to support selection for grain yield stability would enable breeders to streamline evaluation pipelines by reducing the extensive testing of genotypes that show inconsistent or low yields.

Genomic prediction (GP) has become a central tool in plant breeding by leveraging genome-wide marker information to predict complex traits such as yield (Meuwissen et al., 2001; VanRaden, 2008; Endelman, 2011). In soybean, GP models have been successfully applied to improve prediction accuracy and to account for genotype-by-environment interactions (Jarquín et al., 2014a, Jarquín et al., 2014b). However, most GP studies focus on predicting continuous phenotypes, while breeding decisions are inherently discrete. Relatively few studies have explored classification-based GP to support selection decisions. Early work introduced threshold-based models for categorical traits (Montesinos-López et al., 2015), while more recent studies have shown that classification approaches can outperform regression for certain traits and datasets (Merrick et al., 2022; Canella Vieira et al., 2023; Jarquin et al., 2024). Despite these advances, the application of classification-based GP to grain yield and stability in soybean breeding remains limited. This creates a gap between prediction outputs and practical breeding decisions, particularly under early-stage testing constraints.

While research on genotype-by-environment interactions (GEI) and grain yield stability has primarily focused on modeling continuous responses (Jarquín et al., 2014a), classification-based approaches are particularly relevant in breeding because selection decisions are often discrete rather than continuous (e.g., advance vs discard), allowing models to directly optimize decision-making rather than prediction accuracy alone. The objective of this study was to develop and evaluate a classification-based genomic prediction framework for early-stage identification of high-yielding and stable soybean genotypes using a selection index-based decision system that integrates yield performance and consistency across environments into a single, check-referenced metric suitable for early-stage testing.

This approach predicts the likelihood of genotypes belonging to defined yield classes, providing breeders with a practical decision-making tool aligned with their selection thresholds. By prioritizing genotypes with a high probability of superior performance and stability, breeding programs can more effectively allocate limited testing resources on the most promising genotypes, thereby increasing selection accuracy and intensity and ultimately accelerating genetic gain.

## 2 Materials and methods

### 2.1 Data collection

#### 2.1.1 Experimental design and plant materials

A total of 1,789 soybean entries, comprising 1,780 breeding lines developed by the University of Arkansas System – Division of Agriculture Soybean Breeding Program and 9 reference checks, were evaluated for grain yield in multi-environment trials (MET) during 2023 and 2024. These breeding lines were selected from F_4:5_ progeny rows and were derived from 172 bi-parental populations. The evaluated entries included MG III (146), IV (1,544), and V (99).

**Figure 2:**
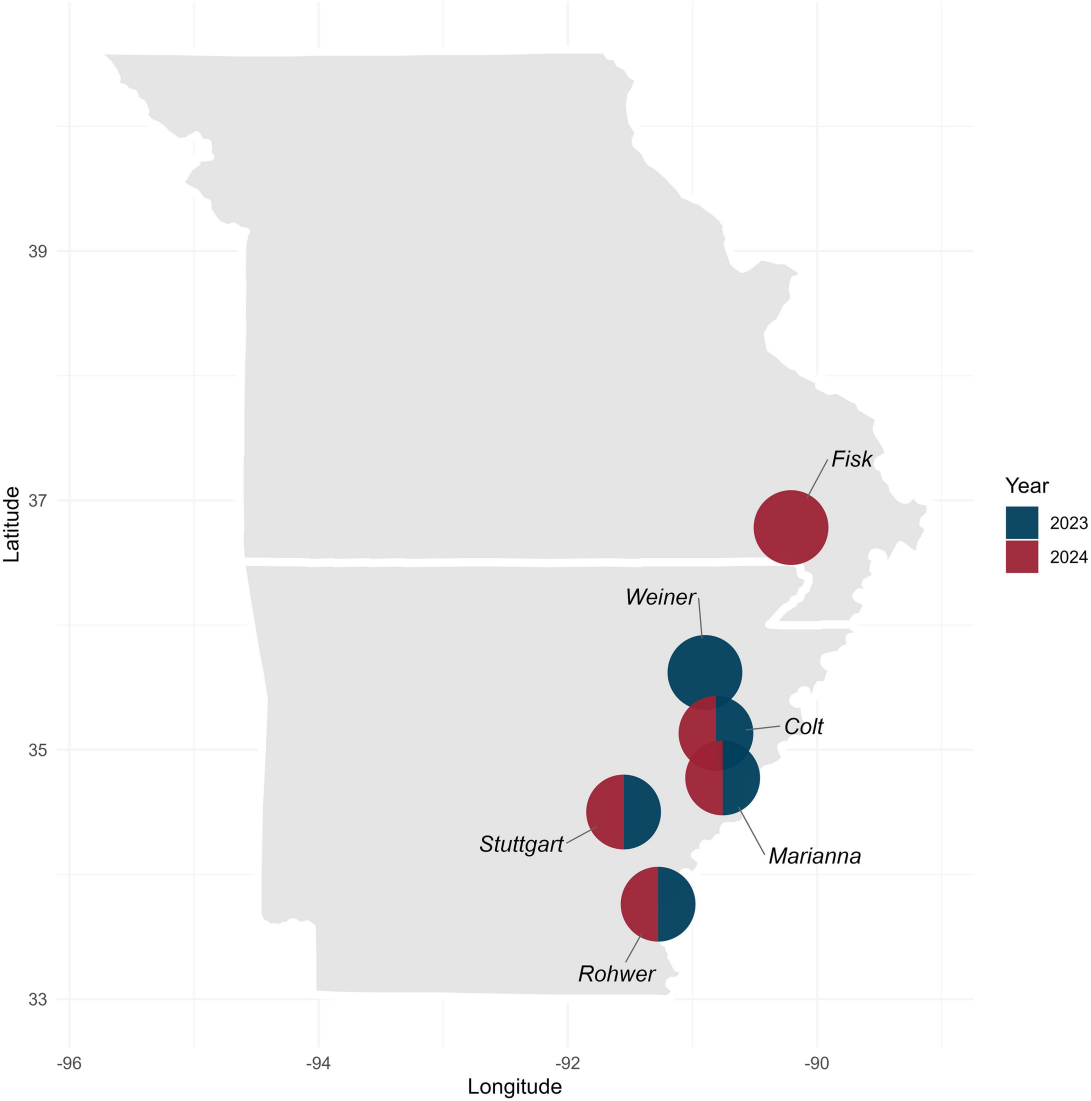
Geographic distribution of soybean yield trial locations in Arkansas and Missouri during 2023 and 2024.

Field trials were conducted using a randomized complete block design (RCBD) with two replications across ten Arkansas environments (year × location combination), during 2023 (5 environments) and 2024 (5 environments) (**Table 1**). Field locations included the Lon Mann Cotton Research Station (LMCRS) in Marianna, AR (34°43’58.31” N, 90°45’59.52” W), the Pine Tree Research Station (PTRS) in Colt, AR (35°07’27.85” N, 90°55’47.60” W), the Rohwer Research Station (RRS) in Rohwer, AR (33°48’37.15” N, 91°16’11.08” W), the Rice Research & Extension Center (RREC) in Stuttgart, AR (34°27’56.1” N, 91°25’20.4” W), Eagle Seeds in Weiner, AR (35°36’55.7” N, 90°53’05.8” W), and MOARK Agricultural Research LLC in Fisk, MO (36°42’43.8” N, 90°12’31.1” W) (**Figure 2**). Field plots consisted of two rows, each 4.00 to 4.67 m long, spaced 0.76 to 0.97 m apart depending on equipment availability at each location.

**Table 1:** Description of early-stage yield trials: year, test entries, locations, and reference checks.

Year	Location^1^	Number of entries	Non-observed^2^	Observed^3^	Reference checks^4^
2023	Marianna	1,789	770	1,019	AG38XF1, AG40XF1, AG43XF2, AG48X9, AG52XF0, AG54XF0, P42A84E, P48A14E, and R18-14502
	Colt		745	1,044	
	Rohwer		1,605	184	
	Weiner		1,605	184	
	Stuttgart		893	896	
2024	Marianna	1,789	929	860	AG38XF1, AG40XF1, AG43XF2, AG48X9, AG52XF0, AG54XF0, P42A84E, P48A14E, and R18-14502
	Fisk		1,708	82	
	Colt		931	858	
	Rohwer		950	839	
	Stuttgart		1,649	140	

^1^ Locations across the state of Arkansas and Missouri, U.S. ^2^Genomic Best Linear Unbiased Prediction (GBLUP). ^3^Grain yield data from the University of Arkansas Soybean Breeding multi-environment trials. ^4^Reference checks, where Asgrow (AG), Pioneer (P), and University of Arkansas (R).

#### 2.1.2 Phenotyping (grain yield and maturity)

Plant maturity was recorded when 95% of the pods in a plot exhibited physiological maturity at the R8 growth stage (Fehr and Caviness, 1977). Grain yield was obtained using a plot combine shortly after reaching physiological maturity. In each year × test, reference checks were included to provide a performance benchmark for the University of Arkansas Soybean Breeding Program. In 2023 and 2024, the commercial checks included AG38XF1, AG40XF1, AG43XF2, AG48X9, AG52XF0, and AG54XF0 (Asgrow) and P42A84E and P48A14E (Pioneer), along with the conventional check R18-14502 (University of Arkansas) (**Table 1**). The herbicide-resistant checks covered a variety of herbicide-tolerance traits, including XtendFlex (dicamba, glyphosate, and glufosinate, 2-amino-4-[hydroxymethylphosphinyl]butanoic acid) and Enlist-E3 (glyphosate, glufosinate, and 2,4-dichlorophenoxyacetic acid).

#### 2.1.3 Genotypic data collection and processing

For DNA extraction, tissue samples of young leaves were collected from plants grown in a greenhouse located in Fayetteville, Arkansas. Samples were taken from each breeding line during the V2-V3 growth stages (Fehr and Caviness, 1977). Young leaves are preferred over older ones for several reasons, including a higher number of nuclei per unit of tissue, fewer secondary metabolites (e.g., phenolics, tannins, polysaccharides), and easier cell disruption. The DNA was extracted using the cetyltrimethylammonium bromide (CTAB) protocol, following standard procedures (Rogers and Bendich, 1985; Allen et al., 2006). DNA quality and concentration were assessed using a BioSpec Nano spectrophotometer (Shimadzu Scientific Instruments Inc., Columbia, U.S.) to confirm the suitability of the samples for genotyping (Richards et al., 1994). Samples with A260/280 ratios of 1.8-2.0, and concentrations ≥ 50 ng µL^-1^ were shipped to the Soybean Genomics and Improvement Laboratory, Beltsville Agricultural Research Center, USDA-ARS, where genotyping was conducted using the SoySNP3K BeadChip (Song et al., 2024), which is a subsample of the SoySNP50K BeadChip (Song et al., 2013). For the application of GBLUP and GP models, single-nucleotide polymorphisms (SNPs) were transformed into numerical values: 0 for the homozygous minor allele, 1 for the heterozygote, and 2 for the homozygous major allele. Monomorphic molecular markers and those with ≥ 20% missing data were removed from the dataset, resulting in a total of 2,479 molecular markers. For the resulting markers with missing data, marker imputation was performed using the R package (R Core Team, 2025) “missForest” (Stekhoven and Bühlmann, 2012). Briefly, this method uses an iterative random forest algorithm to predict missing markers by modeling each marker with missing data as a function of all other markers. The algorithm iteratively updates missing values until convergence, allowing complex nonlinear relationships and interactions among markers to be captured without assuming a specific data distribution.

Genetic similarity among breeding lines was evaluated using principal component analysis (PCA) with the “*prcomp*” function from the “stats” R package (R Core Team, 2025). Concisely, PCA decomposes the SNP variance-covariance matrix, summarizing genomic distances among genotypes into orthogonal axes that capture the major patterns of genetic variation. The first two principal components were used to visualize the genomic distribution of genotypes according to their decision class.

### 2.2 Statistical analysis

#### 2.2.1 Mixed-model analysis

Because not all 1,789 genotypes were evaluated in all ten environments, a genomic linear mixed model was implemented to jointly analyze multi-environment yield data. This approach leverages (i) genomic relationships among genotypes through the genomic relationship matrix (GRM) and (ii) genetic correlations across environments through a factor-analytic (FA) structure (Smith et al., 2005; VanRaden, 2008; Henderson, 1975). This approach is widely used in plant breeding because enables borrowing information across related genotypes and environments, improving prediction accuracy for unobserved genotype × environment combinations.

The GRM was constructed directly from the SNP marker matrix using the method proposed by VanRaden (2008). Let $M\in R^{n\times m}$ denote the marker matrix, where *n* is the number of genotypes and *m* is the number of biallelic markers coded as 0, 1, or 2. Allele frequencies at the marker *j* were calculated as $p_{j}=\bar{M_{\cdot j}}/2$ where $M_{\cdot j}$ is the mean marker score across genotypes. The centered marker matrix was defined as $Z=M-21p^{⊤}$ where 1 is a vector of ones and *p* is the vector of allele frequencies. The GRM was then computed as $G=\frac{ZZ^{⊤}}{2\sum_{j=1}^{m}p_{j}(1-p_{j})}$ which yields a realized additive relationship matrix scaled by the expected marker heterozygosity.

A linear mixed-effects model was fitted using the ASReml-R (VSN International, Hemel Hempstead, UK) package to obtain GBLUPs for each genotype across all tested and untested environments. (Equation 1). Let $y_{lyg}$ denote the BLUE-based yield response of the genotype *g* evaluated in the location *l* during year *y* (Gilmour, 2019). The fitted mixed model was as follows:

(1) $$y_{lyg}=\text{μ}+L_{l}+{\left(L\;:Y\right)}_{ly}+u_{g}+w_{lg}+{\text{ϵ}}_{lyg}$$

Where μ is the overall mean across all genotypes, locations, and years; $L_{l}$ is the main effect of location *l*; ${\left(L\;:Y\right)}_{ly}$ is the location-by-year effect; $u_{g}$is the additive genomic effect of genotype *g*; $w_{lg}$ is the genomic genotype-by-location (GxL) interaction term; and $\epsilon_{lyg}$ is the residual error term.

The vector of additive genomic effects was modeled using the GRM as $u∼N(0,{\text{σ}}_{g}^{2}G)$, where $\sigma_{g}^{2}$is the additive genomic variance and G is the GRM. Genomic GxL effects were modeled using a factor-analytic structure of order 1 (FA1). This FA1 structure captures a dominant latent environmental gradient, allowing genotypes to differ in their sensitivity to environmental conditions while maintaining model parsimony. At the observation level, this was expressed as $w_{lg}={\text{λ}}_{l}f_{g}+{\text{δ}}_{lg}$, where $\lambda_{l}$ is the loading associated with location *l*, describing how location *l* expresses the latent genetic factor; $f_{g}$is the genotype-specific factor score, representing the sensitivity of genotype *g* to the latent location gradient; and $\delta_{lg}$ is an location-specific deviation not explained by the common factor. In matrix form, the FA1 G×L term followed $w∼N(0,({\text{ΛΛ}}^{⊤}+\text{Ψ})⊗G)$ where Λ is an L×1matrix of environment loadings, Ψ is a diagonal matrix containing location-specific genetic variances not captured by the latent factor, ⊗ denotes the Kronecker product, and G propagates genetic covariance across genotypes. This FA parameterization reduces the number of parameters relative to an unstructured covariance model, improving estimation stability and prediction accuracy in multi-environment yield trials.

For the reference checks, genomic information was unavailable; therefore, they were analyzed separately using a mixed model in which genotype effects were modeled without a GRM. A FA1 model was fitted to the genotype-by-location interaction to account for heterogeneous genetic correlations across environments. Because the majority of checks were observed across all (or nearly all) test environments, their BLUPs could be estimated with minimal reliance on genomic relationships, and this is not expected to affect inference for the checks.

The resulting GBLUPs and BLUPs across environments were subsequently used to derive yield stability metrics and to train classification-based genomic prediction models for early-stage selection.

#### 2.2.2 Phenotypic classes

To support early-stage identification of superior soybean genotypes, a selection index (MSI) was calculated based on each genotype’s performance relative to reference checks. For each environment, genotype-specific GBLUPs were standardized as a percentage of the mean yield of the check cultivars within the same MG. To center the scale, the relative yield was subtracted from 100 such that 0 indicates performance equal to the checks’ mean, negative values indicate inferior performance, and positive values indicate superior performance. The MSI for each genotype was then calculated as the average deviation across all ten environments, including both observed and predicted GBLUPs (Equation 2). For example, an MSI value of -5 corresponds to performance equal to 95% of the checks’ mean across environments, whereas an MSI value of 5 corresponds to 105%. To reflect typical decision thresholds in early-stage soybean breeding, genotypes were classified into three yield classes based on their across-environment MSI: high-yielding (MSI ≥ –5), moderate (–5 > MSI ≥ –15), and low-yielding (MSI < –15).

(2) $$MSI_{i}=\frac{1}{n}\sum_{j=1}^{n}\;[(\frac{Y_{ij}}{Check_{j}}\times 100)-100]$$

Where: $MSI_{i}$ represents the average yield deviation of genotype *i* in relation to the reference checks; *n* represents the number of locations where genotype *i* was evaluated; $Y_{ij}$ represents the GBLUPs of genotype *i* in location *j*; $Check_{j}$ represents the average BLUPs of the reference checks in location *j*.

The classification thresholds (-5 and -15) were defined based on typical breeder decision boundaries, representing approximately 95% and 85% of check performance, respectively, which reflect practical advancement criteria in early-stage soybean yield trials. In this stage, breeders are often interested in distinguishing the low-performing genotypes rather than identifying the highest-ranking ones.

#### 2.2.3 Genomic prediction models

Two classification-based GP models, including generalized linear model via elastic net regularization (GLMNet) and random forest (RF), were developed using the R package “*caret”* (Kuhn, 2011). Briefly, GLMNet combines the penalties of lasso and ridge regression to perform variable selection and regularization, thereby enhancing prediction accuracy in high-dimensional datasets, especially when some predictors are more important than others (Zou and Hastie, 2005). RF is an ensemble learning method that builds multiple decision trees using bootstrap samples and averages their predictions to reduce variance and improve generalization (Breiman, 2001). These models differ in their underlying assumptions and decision boundaries, and thus are expected to capture different information from the data. GLMNet emphasizes linear effects and variable selection, isolating the most relevant predictors. RF captures complex non-linear patterns and interactions among variables.

The dataset included 1,789 genotypes (1,780 breeding lines and 9 reference checks) and 2,479 molecular markers, with 298 genotypes in the high-yielding class, 900 in the moderate class, and 582 in the low-yielding class. To train the models, SoySNP3K BeadChip molecular markers were used as predictors, and yield classes were used as the response variable. Model performance was evaluated using a repeated cross-validation framework, implemented with the “*caret”* R package. the dataset was randomly partitioned into 80% training and 20% testing subsets within each repetition using the *createDataPartition* function, with stratified sampling to preserve class proportions across folds. Within each training subset, model tuning and fitting were conducted using 5-fold cross-validation repeated 5 times via the *trainControl* function, ensuring that model hyperparameters were selected exclusively from training data. The held-out 20% testing subset in each repetition was used to generate out-of-fold predictions, which were pooled across repetitions to compute overall performance metrics. This repeated cross-validation framework provides robust estimates of predictive performance while reducing overfitting and sampling variability (Stone, 1978; Kuhn, 2011).

A comprehensive set of classification metrics was calculated to evaluate the usefulness of each model in supporting breeding decisions. Overall accuracy (Equation 3) measured the proportion of correctly classified observations, while the Cohen’s Kappa statistic (Equation 4) assessed agreement between predicted and observed classes beyond chance. Sensitivity (recall) (Equation 5) indicated the ability to correctly identify genotypes within each class, and specificity (Equation 6) measures the proportion of genotypes correctly identified as not belonging to a given class. Precision (Equation 7) measured the proportion of predicted positives that were correctly classified, whereas the negative predictive value (Equation 8) indicated the probability that a predicted negative was correct. The F1-score (Equation 9), defined as the harmonic mean of precision and recall, offered a balanced measure of classification performance. Balanced accuracy (Equation 10), calculated as the average of sensitivity and specificity, accounted for unequal class sizes.

Because the response variable included three yield-performance classes, class-dependent metrics (sensitivity, specificity, precision, negative predictive value, and F1-score) were calculated using a one-versus-rest approach, where each class was considered the positive class and the remaining classes were pooled as the negative class (Hastie et al., 2009). Class-specific values were reported individually and summarized using macro-averaging to ensure equal weighting across classes. Overall accuracy and Cohen’s Kappa were calculated directly from the complete 3 × 3 confusion matrix.

To further evaluate performance under different class distributions, additional metrics were reported: prevalence (Equation 11) (defined as the proportion of observations belonging to a given class), detection rate (Equation 12) (the proportion of the sample correctly identified as positive), and detection prevalence (Equation 13) (the proportion of predicted positives). Confidence intervals (Equation 14) for accuracy were computed using the Wald binomial approximation to quantify sampling variability, and McNemar’s test (Equation 15) was used to assess the statistical significance of differences in paired misclassification rates.

(3) $$Accuracy=\frac{TP+TN}{TP+TN+FP+FN}$$

(4) $$Kappa=\frac{P_{o}-P_{e}}{1-P_{e}}$$

(5) $$Sensitivity(Recall)=\frac{TP}{TP+FN}$$

(6) $$Specificity=\frac{TN}{TN+FP}$$

(7) $$Precision(Positive\;Predictive\;Value)=\frac{TP}{TP+FP}$$

(8) $$Negative\;Predictive\;Value=\frac{TN}{TN+FN}$$

(9) $$F1-Score=\frac{2\times (Precision\times Sensitivity)}{Precision+Sensitivity}$$

(10) $$Balanced\;Accuracy=\frac{Sensitivity+Specificity}{2}$$

(11) $$Prevalence=\frac{TP+FN}{TP+TN+FP+FN}$$

(12) $$Detection\;Rate=\frac{TP}{TP+TN+FP+FN}$$

(13) $$Detection\;Prevalence=\frac{TP+FP}{TP+TN+FP+FN}$$

(14) $$CI_{95\%}=Accuracy\pm 1.96\times \sqrt{\frac{Accuracy(1-Accuracy)}{n}}$$

(15) $${\text{χ}}_{McNemar}^{2}=\frac{{\left(b-c\right)}^{2}}{b+c}$$

Where TP = true positive; TN = true negative; FP = false positive; FN = false negative; $P_{o}$= observed agreement between predicted and observed classes; $P_{e}$= expected agreement by chance; *n* = total number of observations; *b* = number of samples incorrectly classified as positive but actually negative; *c* = number of samples incorrectly classified as negative but actually positive.

To better evaluate the type and frequency of classification errors generated by each model, predictions in the testing set were categorized into three diagnostic groups: correct, mild, and extreme misclassifications. A prediction was labeled correct when the predicted and observed yield classes matched exactly. Mild misclassifications occurred when predictions were one class away from the true label (e.g., low-yielding predicted as moderate). Extreme misclassifications are errors where the predicted class is two classes away from the true label (e.g., low-yielding predicted as high-yielding, and vice-versa). For each model, the number of predictions in each category was calculated and converted into proportions relative to the total number of test observations. These proportions were used to compare the distribution of classification outcomes between models and to quantify the severity of misclassification errors.

## 3 Results

### 3.1 Selection index among breeding lines

The MSI scores ranged from -32.4 to 7.2, with an overall mean of -11.8, resulting in a dataset consisted of 16.7% high-yielding, 50.3% moderate, and 32.5% low-yielding genotypes, indicating a moderate class imbalance typical of early-stage breeding populations. This distribution is consistent with expectations for Preliminary Yield Trials (PYT), where most entries originate from visually selected progeny rows and are therefore predominantly low yielding, with only a small proportion representing superior genotypes. This imbalance highlights the challenge breeders face in allocating limited early-stage testing resources and underscores the potential of GP to better target promising genotypes, thereby increasing selection intensity and accuracy and improving the overall efficiency of advancement decisions.

### 3.2 Prediction accuracy across classification models

To evaluate the effectiveness of classification-based genomic prediction for early-stage selection, GLMNet and RF models were assessed using standard classification metrics derived from confusion matrices based on pooled out-of-fold predictions on the testing set (**Table 2**). These metrics included overall accuracy, balanced accuracy, Cohen’s Kappa, F1-score, negative predictive value, positive predictive value, precision, sensitivity, specificity, and the no-information rate (NIR), along with associated p-values for overall accuracy relative to the no-information rate.

**Table 2:** Summary of confusion matrix evaluation metrics for generalized linear model via elastic net regularization (GLMNet) and random forest (RF) models based on pooled out-of-fold predictions.

Metric	Models	
	GLMNet	RF
95% CI (Lower–Upper)^1^	0.81-0.82	0.80-0.82
Accuracy^2^	0.81	0.81
Balanced Accuracy (Macro)^3^	0.84	0.83
Cohen’s Kappa^4^	0.69	0.68
F1 Score (Macro)^5^	0.80	0.79
McNemar’s Test P-Value^6^	2.42x10^-9^	< 2.20x10^-16^
Negative Predictive Value (Macro)^7^	0.90	0.90
No Information Rate (NIR)^8^	0.51	0.51
Positive Predictive Value (Precision, Macro)^9^	0.81	0.82
P-Value [Acc > NIR]^10^	<2.20x10^-16^	<2.20x10^-16^
Sensitivity (Recall, Macro)^11^	0.78	0.76
Specificity (Macro)^12^	0.89	0.89

^1^ The 95% confidence interval quantifies uncertainty around model accuracy by indicating the range within which the true accuracy is likely to fall. ^2^Accuracy reflects the proportion of genotypes correctly classified across all classes. ^3^Macro balanced accuracy, calculated as the average of sensitivity and specificity, gives equal weight to each class regardless of class imbalance. ^4^Cohen’s Kappa measures agreement between predicted and observed classifications after accounting for chance agreement. ^5^The macro F1 score, defined as the harmonic mean of precision and sensitivity for each class averaged equally, balances false positives and false negatives across classes. ^6^McNemar’s test p-value evaluates whether there is systematic bias in the model’s misclassification toward one class. ^7^The macro negative predictive value represents the average proportion of genotypes predicted as negative that are truly negative across all classes. ^8^The no-information rate represents the baseline accuracy obtained by always predicting the majority class. ^9^The macro positive predictive value reflects the average proportion of predicted positives that are truly positive across all classes. ^10^This p-value tests whether the model’s accuracy is significantly greater than the NIR, indicating performance beyond chance. ^11^Macro sensitivity (recall) measures the average proportion of true positives correctly identified across all classes. ^12^Macro specificity quantifies the average proportion of true negatives correctly identified across all classes.

In terms of overall performance metrics, both RF and GLMNet achieved a high accuracy (0.81), which was substantially greater than the no-information rate (0.51; *p* < 2.2×10–^16^ for both models) (**Table 2**). GLMNet demonstrated slightly higher macro-balanced accuracy (0.84) than RF (0.83) (**Table 2**). Regarding sensitivity, which reflects the model’s ability to correctly identify genotypes across classes, GLMNet achieved the highest macro-averaged value of 0.78, compared with RF at 0.76 (**Table 2**). Conversely, specificity, which measures the ability to accurately identify genotypes not belonging to a given class, was similar for GLMNet and RF (0.89). For positive predictive value, representing the proportion of correctly classified genotypes among those predicted to belong to a class, RF achieved the highest macro-averaged value (0.82), followed by GLMNet (0.81) (**Table 2**). Both RF and GLMNet exhibited a high macro-averaged negative predictive value (0.90), indicating a strong ability to correctly identify genotypes that do not belong to a given yield class.

To assess whether models favored the majority class by chance, Cohen’s Kappa coefficients indicated substantial agreement beyond chance for both GLMNet (0.69) and RF (0.68), demonstrating that both models capture meaningful signal rather than reflecting class prevalence alone (**Table 2**).

The class-specific diagnostic metrics for GLMNet and RF provide a detailed assessment of each model’s performance within each class (**Table 3**). GLMNet showed higher detection of both low- and high-yielding genotypes, as reflected in its higher sensitivity and balanced accuracy for these classes (**Table 3**). In contrast, RF demonstrated the highest sensitivity for the moderate class (0.88), but had lower sensitivity for both low- and high-yielding classes compared to GLMNet, indicating a reduced ability to distinguish between these groups, despite slightly higher overall macro-averaged precision.

**Table 3:** Performance metrics by yield class for Generalized Linear Model via Elastic Net Regularization (GLMNet) and Random Forest (RF) classification models.

Model	Class	Balanced accuracy^1^	Sensitivity^2^	Specificity^3^	Positive predictive value ^4^	Negative predicted value^5^	F_1_-score^6^
GLMNet	Low-Yielding	0.89	0.85	0.93	0.86	0.93	0.85
	Moderate	0.82	0.84	0.79	0.80	0.83	0.82
	High-Yielding	0.81	0.66	0.96	0.76	0.93	0.71
RF	Low-Yielding	0.89	0.82	0.95	0.89	0.92	0.85
	Moderate	0.81	0.88	0.74	0.78	0.86	0.83
	High-Yielding	0.78	0.59	0.97	0.79	0.92	0.68

^1^ Balanced accuracy, calculated as the average of sensitivity and specificity for each class under a one-versus-rest framework, accounting for unequal class sizes. ^2^Sensitivity (recall), defined as the proportion of true positives correctly identified for the focal class. ^3^Specificity, defined as the proportion of true negatives correctly identified for the focal class. ^4^Positive predictive value (precision), defined as the proportion of predicted positives that are truly positive for the focal class. ^5^Negative predictive value, defined as the proportion of predicted negatives that are truly negative for the focal class. ^6^F1-score, defined as the harmonic mean of precision and sensitivity for the focal class.

Overall, GLMNet showed more balanced classification across the three yield classes, outperforming RF in macro-averaged balanced accuracy, sensitivity, and F1-score (**Table 3**). In contrast, RF exhibited slightly higher macro-averaged precision, while overall accuracy and negative predictive value were similar between models. Despite minor differences, both models demonstrated meaningful predictive ability, primarily when used to eliminate poor candidates from further yield trials. These results suggest that combining classification-based GP with a selection index may help breeders prioritize superior genotypes for evaluation prior to designing their early-stage yield trials.

### 3.3 Misclassification severity across models

Across both models, most predictions were correct for GLMNet and RF (81%), and most errors were mild (18% and 19%, respectively), indicating that misclassifications rarely involved confusion between the low- and high-yielding extremes (both 0%). RF demonstrated a slightly higher proportion of mild classifications than GLMNet, consistent with its tendency to assign ambiguous genotypes to the intermediate moderate class, resulting in a more conservative error profile. The predominance of correct classifications, along with mild rather than extreme errors, suggests that classification-based GP provides a practical and relatively low-risk framework for supporting early-stage advancement decisions in soybean breeding.

## 4 Discussion

Breeding programs are often referred to as a numbers game, which requires an efficient balance between resources and the integration of diverse knowledge, tools, and expertise (Vieira and Chen, 2021). In soybean breeding, early-stage yield trials are constrained by limited seed availability and the large number of candidate genotypes, making it challenging to accurately evaluate grain yield and stability across diverse environments (**Figure 1**). Efficient approaches that identify high-yielding and stable genotypes early in the breeding cycle are therefore essential. In this study, we propose a classification-based GP framework designed to improve resource allocation by accurately discarding undesirable (low-yielding and/or unstable) genotypes before advancing them to larger and more costly MET. By eliminating inferior genotypes early, breeders can either reduce overall testing costs or reallocate the same level of resources toward evaluating more environments or more genotypes at later stages (Persa et al., 2023), ultimately increasing MET efficiency and accelerating genetic gain through improved selection intensity and accuracy.

Across many soybean breeding programs, early-generation advancement often produces large pools of germplasm where numerous low-yielding lines are retained, while only a small fraction are truly superior (Diers et al., 2018). This reduces selection intensity and increases testing costs (Escamilla et al., 2025). These challenges fit within the “breeding funnel” concept: a broad base of diversity in the early stages gradually narrows through successive years of testing, while the number of locations increases. Under this framework, inferior genotypes must be identified and removed as early as possible, allowing resources to be focused on the most promising candidates and improving overall breeding efficiency per cycle (Botega et al., 2025).

One practical tool for identifying superior genotypes prior to extensive MET is the use of genomic prediction (GP) (Vieira et al., 2025). While conventional GP focuses on predicting continuous phenotypes, classification-based GP directly addresses categorical breeding decisions, such as advance vs. discard, or resistant vs. susceptible in the case of resilience to biotic and abiotic stresses (Mbebi et al., 2025). Several studies demonstrate the value of classification-based GP for complex traits. For example, both RF and support vector machine (SVM) have performed well for southern root-knot nematode [*Meloidogyne incognita* (Kofold & White) Chitwood] resistance in soybean (Canella Vieira et al., 2022), and classification approaches have been useful for morphological traits such as seed coat color, pod color, and pubescence density (Gill et al., 2022). These results emphasize the potential of classification-based models to support genotype characterization and decision-making across breeding pipelines.

When working with complex traits, such as grain yield stability, ML algorithms can capture complex, nonlinear, and high-order interactions among markers without requiring prior specification of the genetic architecture. This flexibility is particularly advantageous when the number of predictors far exceeds the number of observations (p > n) (Crossa et al., 2025). This approach has gained significant traction across various breeding efforts, and previous studies highlight the advantages of ML and classification frameworks for improving selection accuracy. Heslot et al. (2012) showed that nonparametric models, especially RF, capture non-additive genetic effects missed by linear methods. Ornella et al. (2014) further demonstrated that reframing GP as a classification problem improves selection accuracy, reporting that SVM and RF achieved higher Cohen’s Kappa and greater relative efficiency for identifying the top 15% of genotypes across diverse maize and wheat datasets. These studies support the use of classification-based algorithms for improving selection outcomes in breeding programs.

A recurring challenge in classification problems is class imbalance. In crop breeding data, where the majority of genotypes are low-yielding and only a small proportion are superior, ML models can be biased toward the majority class (Provost, 2000; Cieslak and Chawla, 2008). This issue is well documented across big data applications in agriculture and beyond (Kumar et al., 2021). In this study, although class-specific metrics varied across models and classes, the observed performance levels are consistent with expectations for multi-class GP under substantial class imbalance. Yield-class boundaries inherently compress phenotypic variability in soybean breeding programs, leaving the minority class underrepresented, making sensitivity notoriously challenging to achieve. Despite these constraints, GLMNet and RF maintained macro-balanced accuracy above 0.80 and produced uniform precision and negative predictive value across classes, indicating reliable discrimination and low false-negative rates (**Tables 2**, **3**). RF demonstrated stronger detection of the moderate genotypes (**Tables 2**, **3**). These results suggest that both models performed well in classifying genotypes under imbalanced scenarios and are well-suited for the implementation in soybean breeding programs for the early identification of superior genotypes.

In terms of overall metrics, the slightly superior performance of GLMNet over RF may be due to the combination of L1 and L2 penalties to keep important markers while shrinking the effects of less relevant ones, which improves generalization when many predictors have minor effects (Zou and Hastie, 2005; Ogutu et al., 2012). RF, in contrast, is a tree-based method designed to capture complex non-linear relationships and high-order interactions among markers (Breiman, 2001). Still, RF conservative decision boundaries may reduce sensitivity for extreme yield classes. This trade-off highlights an important distinction between model types: while penalized regression methods such as GLMNet emphasize generalization through shrinkage of marker effects, tree-based methods such as RF prioritize capturing non-linear interactions but may produce more conservative class boundaries. The choice between these approaches should therefore consider the breeding objective, particularly whether minimizing extreme errors or maximizing detection of superior genotypes is prioritized.

**Figure 3:**
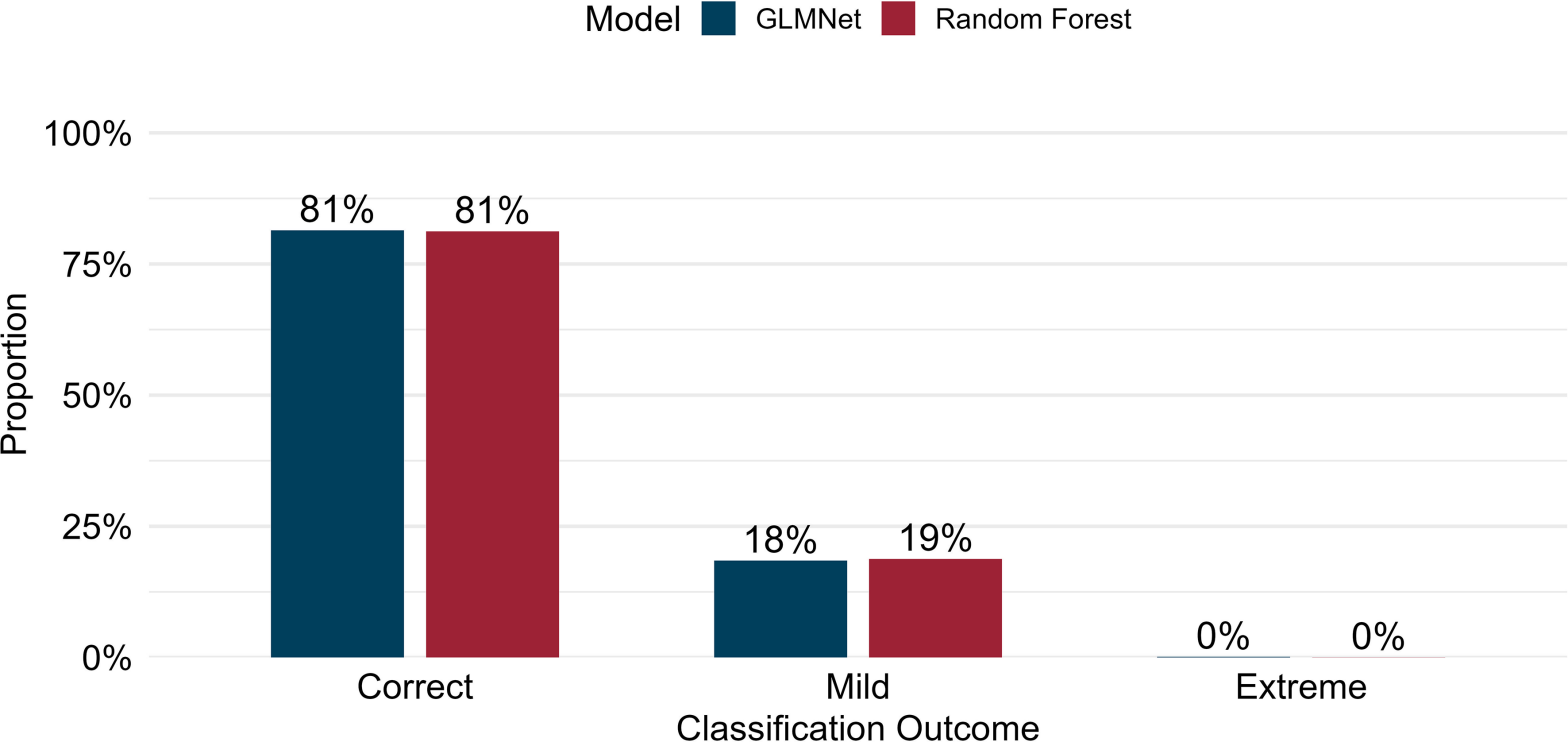
Proportion of correct, mild, and extreme misclassification outcomes for GLMNet and Random Forest models based on pooled out-of-fold predictions across repeated cross-validation. Correct classifications correspond to predictions matching the observed yield class. Mild misclassifications represent predictions that were one class away from the true label (e.g., low-yielding predicted as moderate). Extreme misclassifications represent errors where the predicted class is two classes away from the true label (e.g., low-yielding predicted as high-yielding, and vice-versa). Percentages indicate the proportion of total predictions falling into each category.

Herein, most classifications were accurate across both models (**Figure 3**). The majority of misclassifications were mild, where the predicted class differed by one category from the true label, while extreme errors were rare (**Figure 3**). This pattern highlights the potential of both algorithms to support early-stage breeding decisions, especially when limited seed availability and the high number of genotypes present challenges for testing capacity. Interestingly, although GLMNet showed a slight advantage over RF in class-specific metrics, RF slightly outperformed GLMNet when misclassification types were compared (**Figure 3**). This result is likely due to RF’s conservative decision boundaries, which tend to assign ambiguous genotypes to the intermediate “moderate” class, thereby reducing severe errors. Distinguishing between correct, mild, and extreme errors provides more actionable insights for breeders than traditional confusion-matrix metrics, as each error type has different practical consequences. Mild errors, for example, misclassifying a low-yielding genotype as moderate, still allow inferior lines to be detected during early-stage yield trials. In contrast, extreme errors such as predicting a high-yielding genotype as low-yielding may prematurely eliminate elite candidates and impose substantial strategic costs. However, this risk was minimal in the present study, as across all pooled out-of-fold predictions from cross-validation, only three instances of high-yielding genotypes were misclassified as low-yielding by GLMNet, and none by RF, representing <0.01% of the total prediction set (data not shown). The extreme rarity of these high-cost errors across both models supports their use as decision-support tools in early-stage breeding, where large numbers of genotypes and limited seed availability constrain the accurate assessment of grain yield.

**Figure 4:**
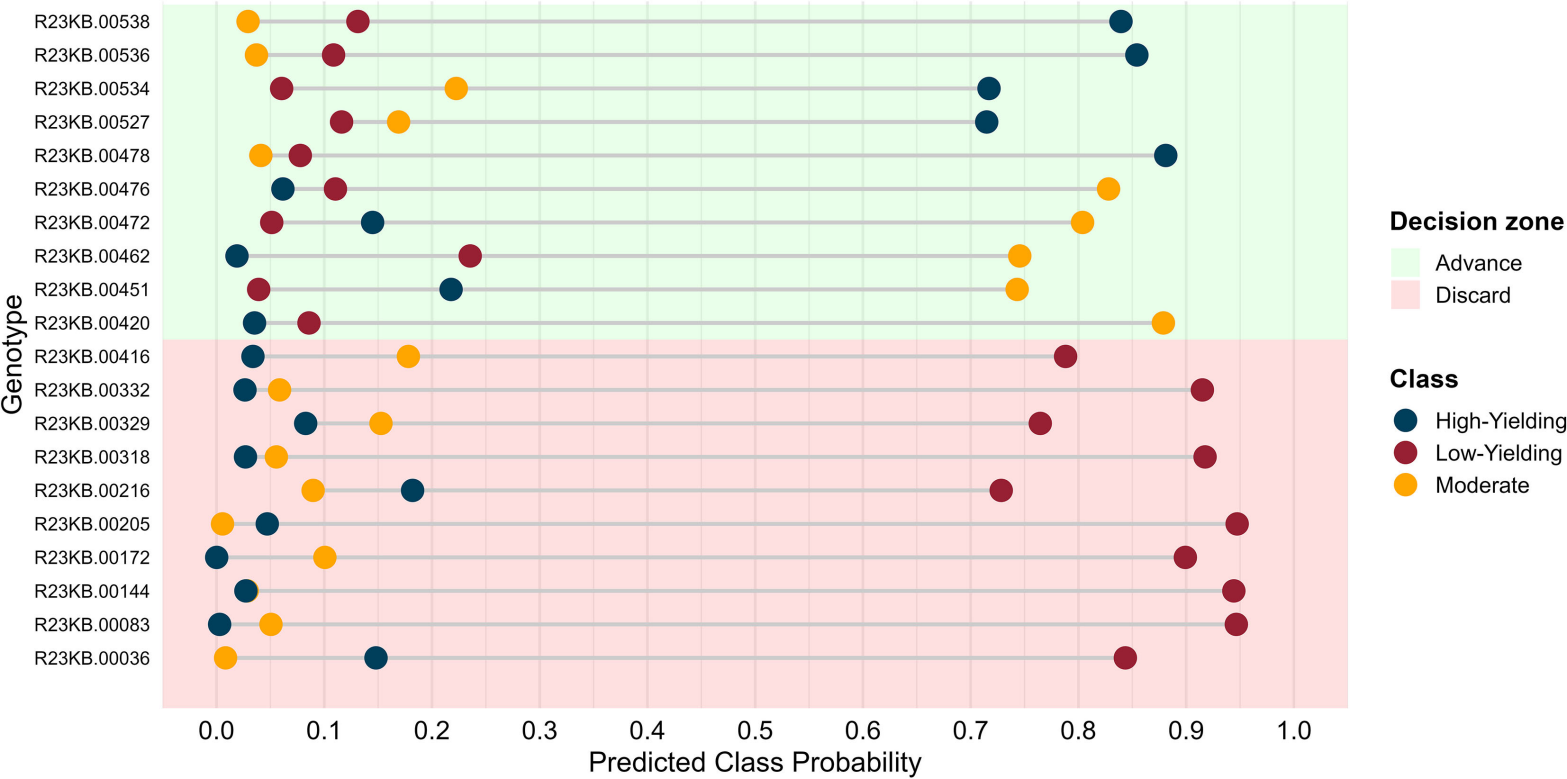
Example of genotype pre-screening using predicted MSI-class probabilities to guide advancement decisions under limited testing capacity. The figure shows a representative subset of genotypes selected to illustrate how predicted class probabilities interact with MSI-based decision zones. Background shading denotes advancement (green) and discard (red) regions defined *a priori* based on classification thresholds (e.g., genotypes with a predicted probability ≥ 0.70 of belonging to the low-yielding class were designated for discard). Points represent predicted class probabilities for individual genotypes, colored by assigned yield class. The same decision rules were applied to all 1,780 genotypes, although only a subset is shown for visualization clarity.

The use of predicted probabilities provides an additional layer of decision-making that is directly relevant to breeding (Montesinos López et al., 2022) (**Figure 4**). Instead of relying solely on class labels, breeders can set probability thresholds to discard genotypes with high confidence of belonging to the low-yielding class (**Figure 4**). For example, using a decision threshold of 0.70 for the low-yielding class, our analysis showed that approximately 40% of all genotypes could be confidently discarded before yield trials (data not shown). This approach enhances selection intensity by removing inferior lines early, while explicitly managing the trade-off between the risk of discarding promising material and the cost of testing poor performers. The use of classification-based predictions further aligns with practical breeding decisions, which are inherently categorical (e.g., advance vs discard). Compared to regression-based approaches, classification simplifies decision-making and reduces sensitivity to small phenotypic differences, although it may sacrifice resolution in distinguishing among top-performing genotypes.

To assess whether proposed discard decisions were driven primarily by genetic relatedness, we examined the genomic distribution of genotypes proposed for advancement or discard using a PCA (**Supplementary Figure 2**). While genotypes proposed for discard show some clustering within specific regions of the genetic space, the substantial overlap between proposed advance and discard groups indicates that model predictions are not explained solely by overall genetic similarity (**Supplementary Figure 2**). The presence of a clustered group of discarded genotypes is consistent with classical breeding theory, in which crosses between elite parents (“*good x good*”) may nonetheless generate progeny with inferior performance due to unfavorable allele combinations arising from recombination. Although parental lines may exhibit high yield potential, recombination can assemble allele combinations that are suboptimal for yield, leading to coherent groups of related but low-performing progeny within the genetic space (Bernardo, 2020). Importantly, genome-wide relatedness can capture broad yield potential, yet fail to reflect the influence of a small number of high-effect loci controlling stress-resilience responses. Under conditions in which these loci are expressed, differences in performance among otherwise closely related genotypes may be driven by a few critical marker effects rather than by overall genetic similarity (Rupe et al., 2000; Cox et al., 2018; Canella Vieira et al., 2021).

Across selection intensities, GBLUP, GLMNet, and RF differed in how they balanced selection for yield with maintenance of genetic diversity (**Supplementary Table 1**). Agreement among models in identifying top-yielding genotypes increased as selection intensity relaxed, indicating that superior performance is driven by genetic signals that are robust to model choice (**Supplementary Figure 3**). Despite this convergence, PCA-based diversity metrics showed that all models concentrated high-performing genotypes into narrower genomic regions relative to the unselected population, consistent with directional selection toward favorable allelic backgrounds (**Supplementary Table 1**). Among high-yielding lines, GBLUP retained slightly greater genomic diversity than RF and GLMNet, particularly at broader selection thresholds, suggesting a more balanced ability to identify high-yielding while minimizing diversity loss (**Supplementary Figure 4**).

In contrast, greater model divergence was observed when identifying low-yielding genotypes (**Supplementary Figure 3**). RF retained greater genetic diversity by discarding low-yielding genotypes, as indicated by greater PCA dispersion and lower contraction of genomic diversity relative to the remaining population (**Supplementary Table 1**). This pattern suggests that RF preferentially removes underperforming lines while preserving a broader representation of genetic backgrounds (**Supplementary Figure 4**). In contrast, GBLUP tends to discard a wider range of low-yielding genotypes, reflected in diversity ratios closer to or below those of the remaining population, which may increase the risk of prematurely narrowing the genetic base in early-stage yield trials (**Supplementary Table 1**). GLMNet showed intermediate behavior, balancing the removal of low-yielding genotypes with moderate preservation of diversity (**Supplementary Figure 4**). Overall, these results suggest that RF may be better suited to eliminating low-yielding genotypes while maintaining broader genetic diversity, whereas GBLUP requires more cautious use to avoid unintended loss of potential genetic diversity. These differences among models underscore a trade-off in breeding between selection intensity and maintenance of genetic diversity. While aggressive removal of low-performing genotypes can increase short-term gain, it may also reduce long-term genetic variability, which is critical for sustained breeding progress.

Linkage disequilibrium (LD) decay was evaluated to assess whether the marker density used for genomic prediction adequately captures genome-wide genetic relationships while preserving sufficient recombination signal for long-term selection. Linkage disequilibrium decayed rapidly within approximately 1 Mb and stabilized at low r² values (approximately 2–3 Mb), indicating recombination and residual background LD typical of elite soybean breeding germplasm (**Supplementary Figure 5**). This decay profile suggests that current marker densities are adequate to capture most additive genetic variation for genomic-based prediction models while maintaining sufficient recombination potential to sustain long-term genetic gain.

Together, these findings demonstrate that combining MSI with classification-based GP allows breeders to accurately identify superior genotypes prior to large-scale MET while maintaining genetic diversity. Implementing these tools can substantially improve resource allocation in early-stage testing, enabling breeders to focus limited capacity on genotypes with the greatest potential for yield and stability.

## 5 Conclusions

Our results support the use of a classification-based genomic prediction framework to improve early-stage selection in soybean breeding. By integrating machine learning classifiers with an MSI-based evaluation system, this approach provides a practical strategy to identify and remove low-performing genotypes prior to preliminary yield trials. In this study, applying a predicted-probability threshold of 0.7 for the low-yielding class allowed the removal of approximately 40% of genotypes while maintaining low rates of extreme misclassification, supporting more efficient allocation of limited testing resources. Both GLMNet and RF showed consistent predictive ability, indicating their utility for early advancement decisions. However, genotypes were evaluated under sparse testing conditions, where many genotype × environment combinations are unobserved and must be predicted. As a result, classification outcomes depend on the accuracy of genomic predictions and the MSI framework used to summarize performance across environments. In addition, class imbalance remains a limitation, with lower sensitivity for the high-yielding class potentially reducing the ability to identify rare superior genotypes. These results are based on a specific population, set of environments, and MSI-based classification thresholds, and may not directly generalize to other breeding programs or traits. Overall, this framework provides a decision-oriented approach that aligns genomic prediction with interpretable breeding objectives and can be used as a filtering step to reduce unnecessary early-stage testing. Its broader application should be evaluated within the context of population structure, trait architecture, and available training data.

## Data Availability

The datasets presented in this article are not readily available because they include proprietary breeding germplasm and associated data that are subject to intellectual property and programmatic restrictions. Data may be made available upon reasonable request for research purposes, subject to institutional approval and a data use agreement. Requests should be directed to Caio Canella Vieira at caioc@uark.edu.

## Supplementary material

Supplementary Figure 1 Distribution of observed and predicted genotype-by-environment combinations in 2023 and 2024. Each horizontal bar represents an environment within a given year, and each vertical segment corresponds to a genotype. Blue segments indicate genotype-by-environment combinations with observed phenotypic data, whereas red segments indicate combinations for which phenotypes were unobserved and subsequently predicted by the model. Environment abbreviations correspond to yield trial locations: COL, Colt; AR; FIS, Fisk; MO; MAR, Marianna; AR; ROH, Rohwer; AR; STU, Stuttgart; AR; WEI, Weiner; AR.

Supplementary Figure 2 Principal component analysis (PCA) of genomic marker data showing the genetic space of soybean genotypes classified as advance or discard based on averaged out-of-fold prediction probabilities from the GLMNet model. Genotypes with a predicted probability ≥ 0.70 of belonging to the low-yielding class were classified as discard, while all others were classified as advance.

Supplementary Figure 3 Overlap among genomic prediction models in identifying high- and low-yielding soybean genotypes across selection intensities (Top and Bottom 10%, 20%, and 30%). Bars show the proportion of shared genotypes between pairs of models (GBLUP, RF, and GLMNet) and across all three models, based on averaged prediction scores per genotype and model. Higher overlap indicates stronger agreement among models in identifying superior or inferior-yielding lines, whereas lower overlap reflects model-specific selection patterns.

Supplementary Figure 4 Principal component analysis (PCA) of genomic marker data showing the genetic distribution of soybean genotypes selected as high-yielding according to averaged genomic prediction scores from three models (GBLUP, RF, and GLMNet). Colored points represent selected genotypes at three selection intensities (Top or Bottom 10%, 20%, and 30%), while gray points indicate the remaining unselected population.

Supplementary Figure 5 Principal component analysis (PCA) of genomic marker data showing the genetic distribution of soybean genotypes selected as low-yielding according to averaged genomic prediction scores from three models (GBLUP, RF, and GLMNet). Colored points represent selected genotypes at three selection intensities (Top or Bottom 10%, 20%, and 30%), while gray points indicate the remaining unselected population.

Supplementary Figure 6 Linkage disequilibrium (LD) decay estimated from genome-wide marker from breeding lines. Pairwise LD (r²) is plotted against physical genetic distance (Mb), with points representing average LD within distance bins and the solid line indicating the fitted decay trend.

Supplementary Table 1 Genetic diversity analysis of top- and bottom-yielding selected genotype sets relative to the unselected population.^a^Selection indicates whether genotypes were selected from the highest-performing (“Top”) or lowest-performing (“Bottom”) fraction based on predicted yield. ^b^Cutoff represents the proportion of genotypes selected (e.g., Top 10% or Bottom 20%). ^c^Average predicted MSI value (GBLUP) and average predicted probability of being high-yielding (GLMNet and RF) to each selected subset across methods. ^d^PCA Dispersion Ratio is the relative spread of selected genotypes in genomic principal component space compared with the unselected population; values <1 indicate reduced diversity, whereas values ≥ 1 indicate similar or broader diversity. ^e^PCA Coverage Ratio reflects the proportion of the total genomic PCA space occupied by the full population that is represented by the selected subset, providing a measure of genetic breadth. ^f^Genomic Relationship Matrix (GRM) Diversity Ratio represents the relative genetic diversity based on the GRM, calculated as 1 minus mean relatedness; higher values indicate greater genetic diversity retained among selected genotypes relative to the unselected population.
