# Supplementary figures and images for: Classification-based genomic prediction for early identification of high-yielding and stable soybean genotypes
Source: Front Plant Sci. 2026 Apr 29;17:1770360. doi: 10.3389/fpls.2026.1770360 (PMC13168174; doi:10.3389/fpls.2026.1770360)

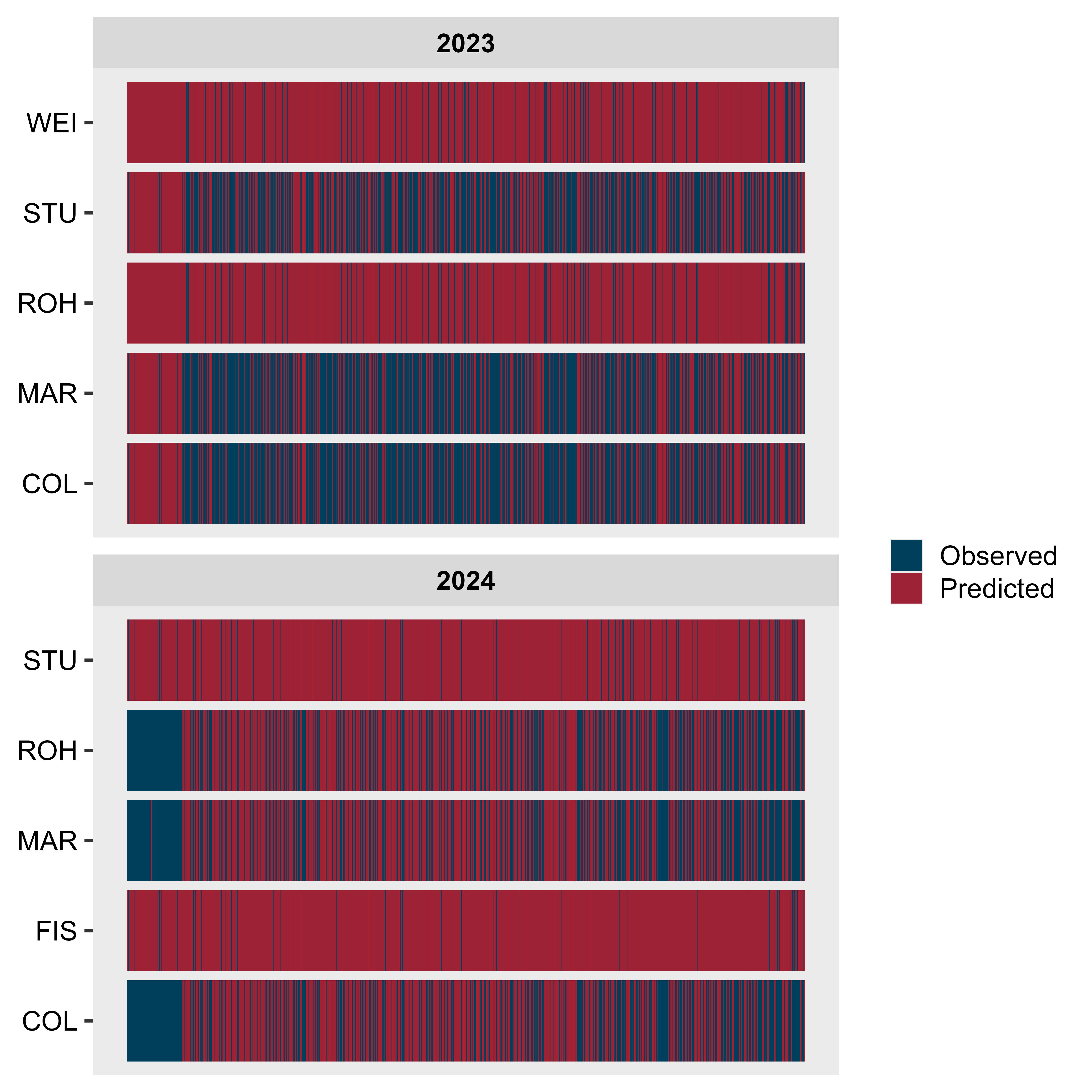

Supplement: Supplementary Figure 1 — Distribution of observed and predicted genotype-by-environment combinations in 2023 and 2024. Each horizontal bar represents an environment within a given year, and each vertical segment corresponds to a genotype. Blue segments indicate genotype-by-environment combinations with observed phenotypic data, whereas red segments indicate combinations for which phenotypes were unobserved and subsequently predicted by the model. Environment abbreviations correspond to yield trial locations: COL, Colt; AR; FIS, Fisk; MO; MAR, Marianna; AR; ROH, Rohwer; AR; STU, Stuttgart; AR; WEI, Weiner; AR. [file Image1.jpeg]

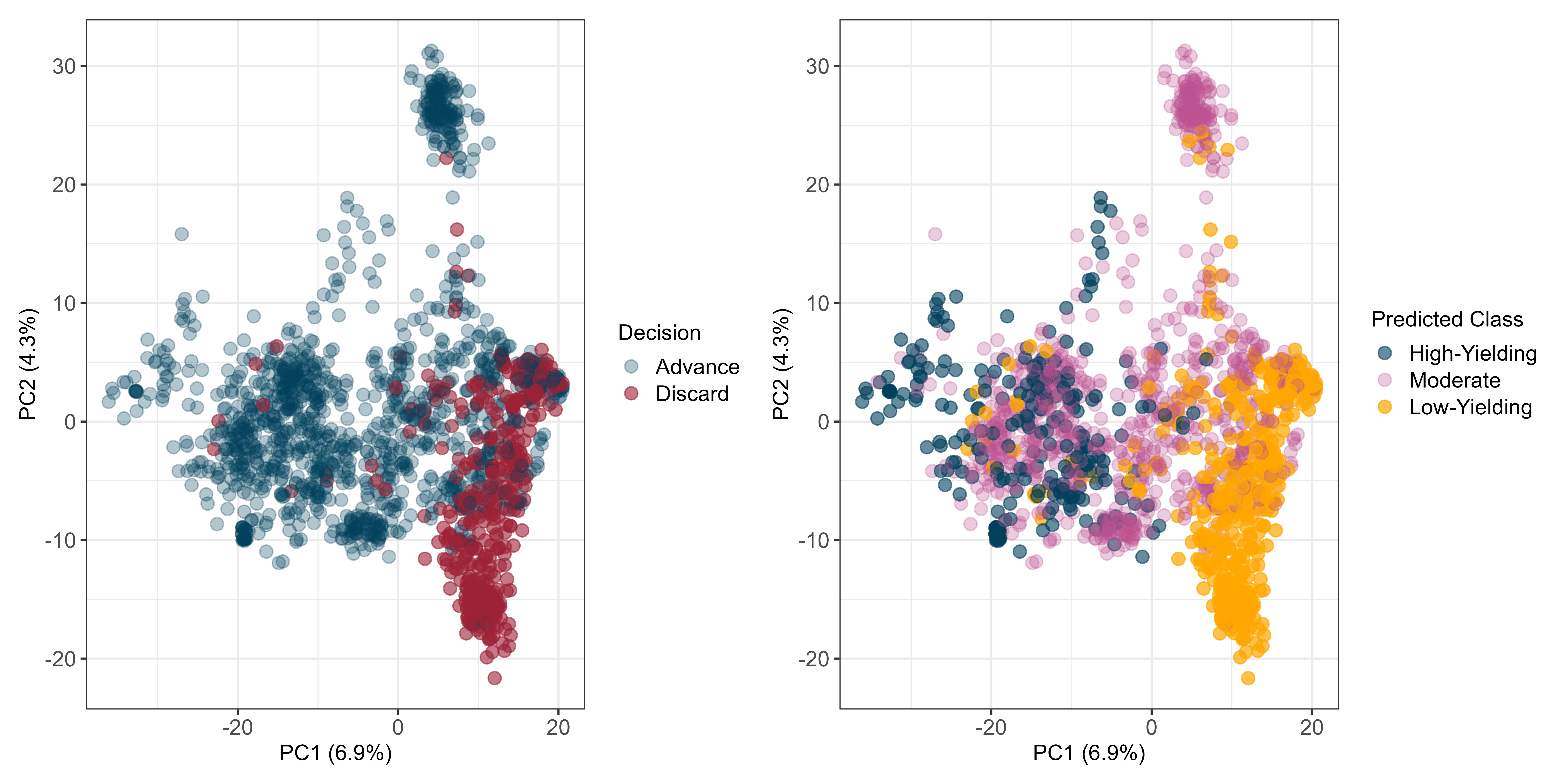

Supplement: Supplementary Figure 2 — Principal component analysis (PCA) of genomic marker data showing the genetic space of soybean genotypes classified as advance or discard based on averaged out-of-fold prediction probabilities from the GLMNet model. Genotypes with a predicted probability ≥ 0.70 of belonging to the low-yielding class were classified as discard, while all others were classified as advance. [file Image2.jpeg]

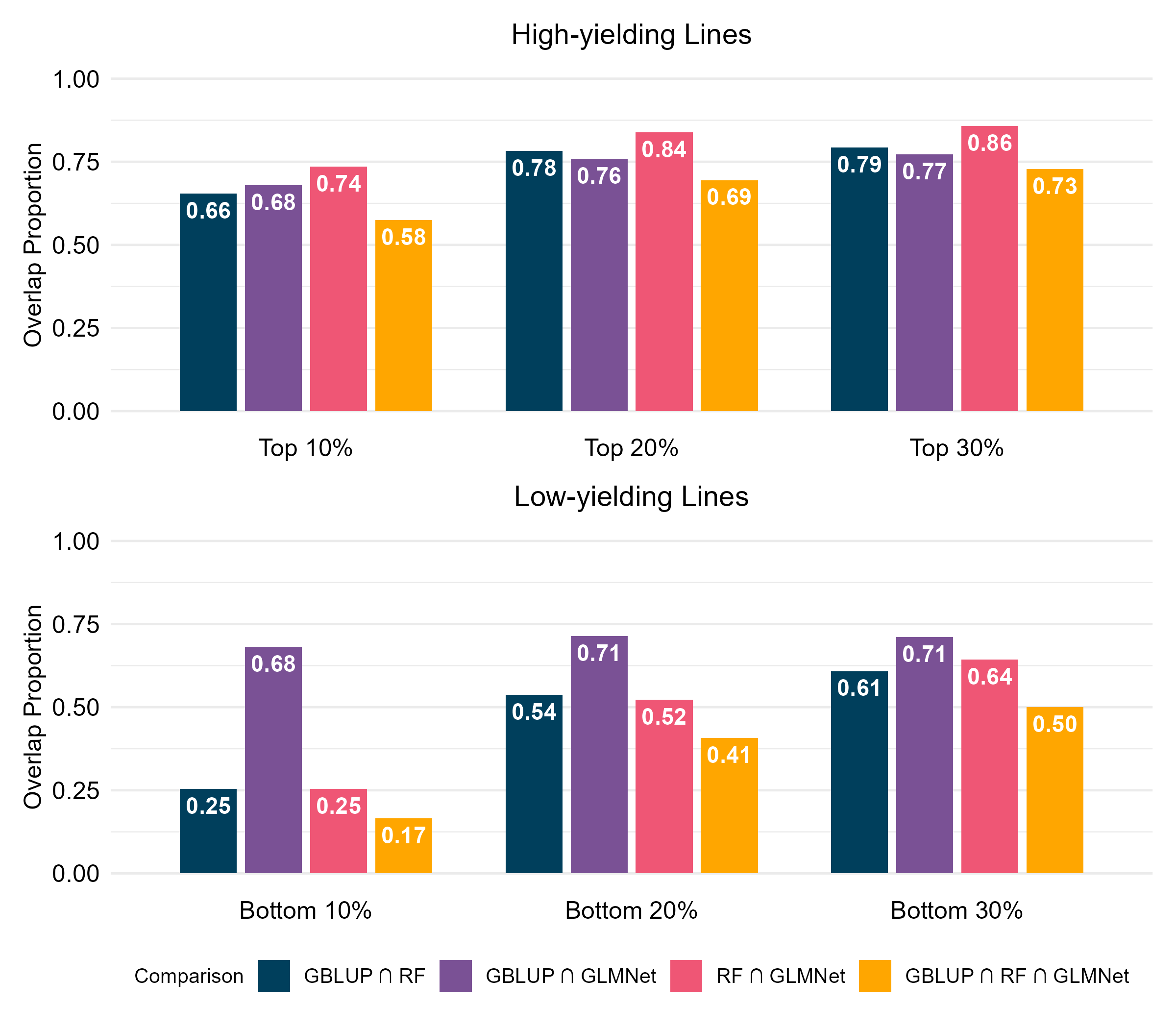

Supplement: Supplementary Figure 3 — Overlap among genomic prediction models in identifying high- and low-yielding soybean genotypes across selection intensities (Top and Bottom 10%, 20%, and 30%). Bars show the proportion of shared genotypes between pairs of models (GBLUP, RF, and GLMNet) and across all three models, based on averaged prediction scores per genotype and model. Higher overlap indicates stronger agreement among models in identifying superior or inferior-yielding lines, whereas lower overlap reflects model-specific selection patterns. [file Image3.png]

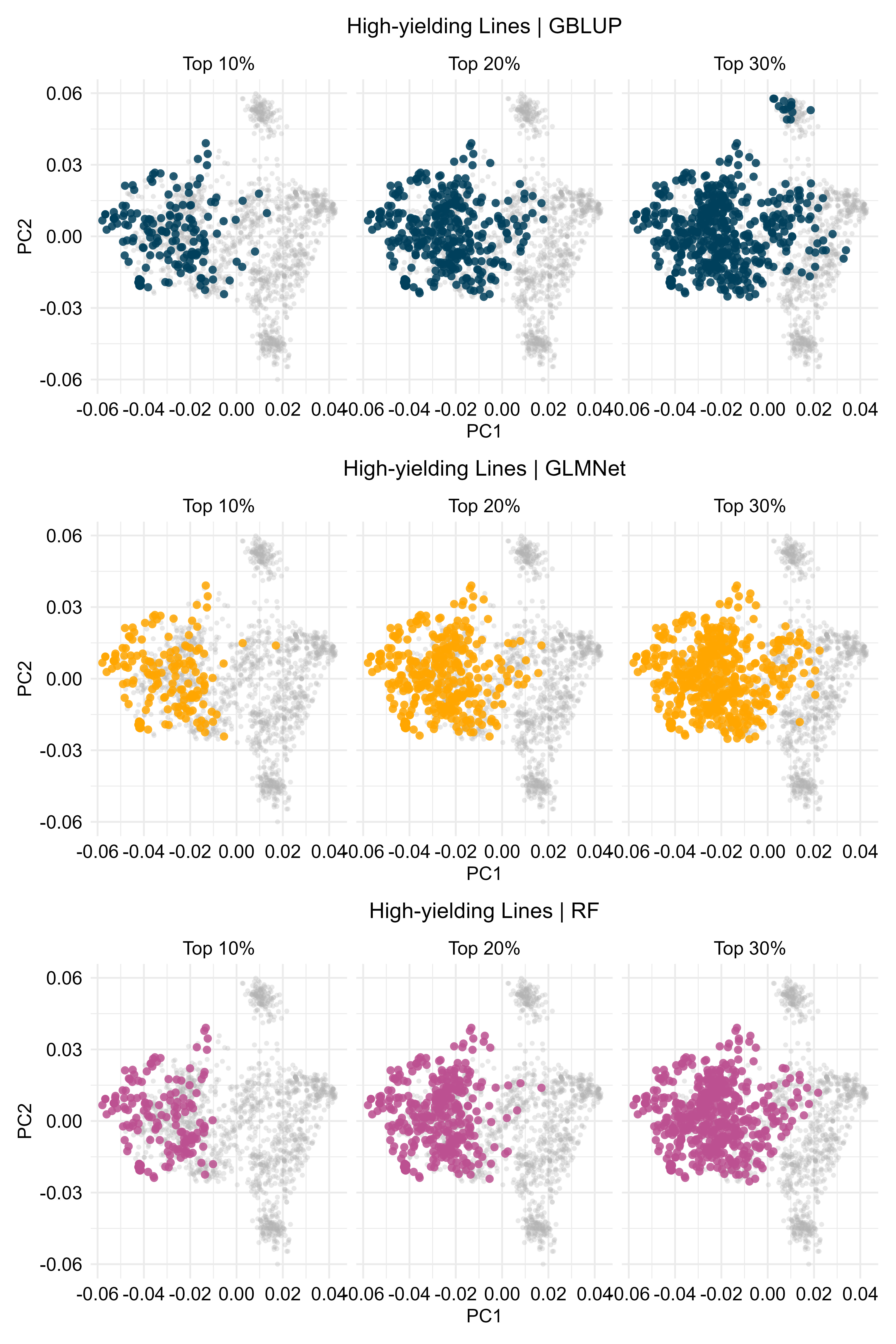

Supplement: Supplementary Figure 4 — Principal component analysis (PCA) of genomic marker data showing the genetic distribution of soybean genotypes selected as high-yielding according to averaged genomic prediction scores from three models (GBLUP, RF, and GLMNet). Colored points represent selected genotypes at three selection intensities (Top or Bottom 10%, 20%, and 30%), while gray points indicate the remaining unselected population. [file Image4.jpeg]

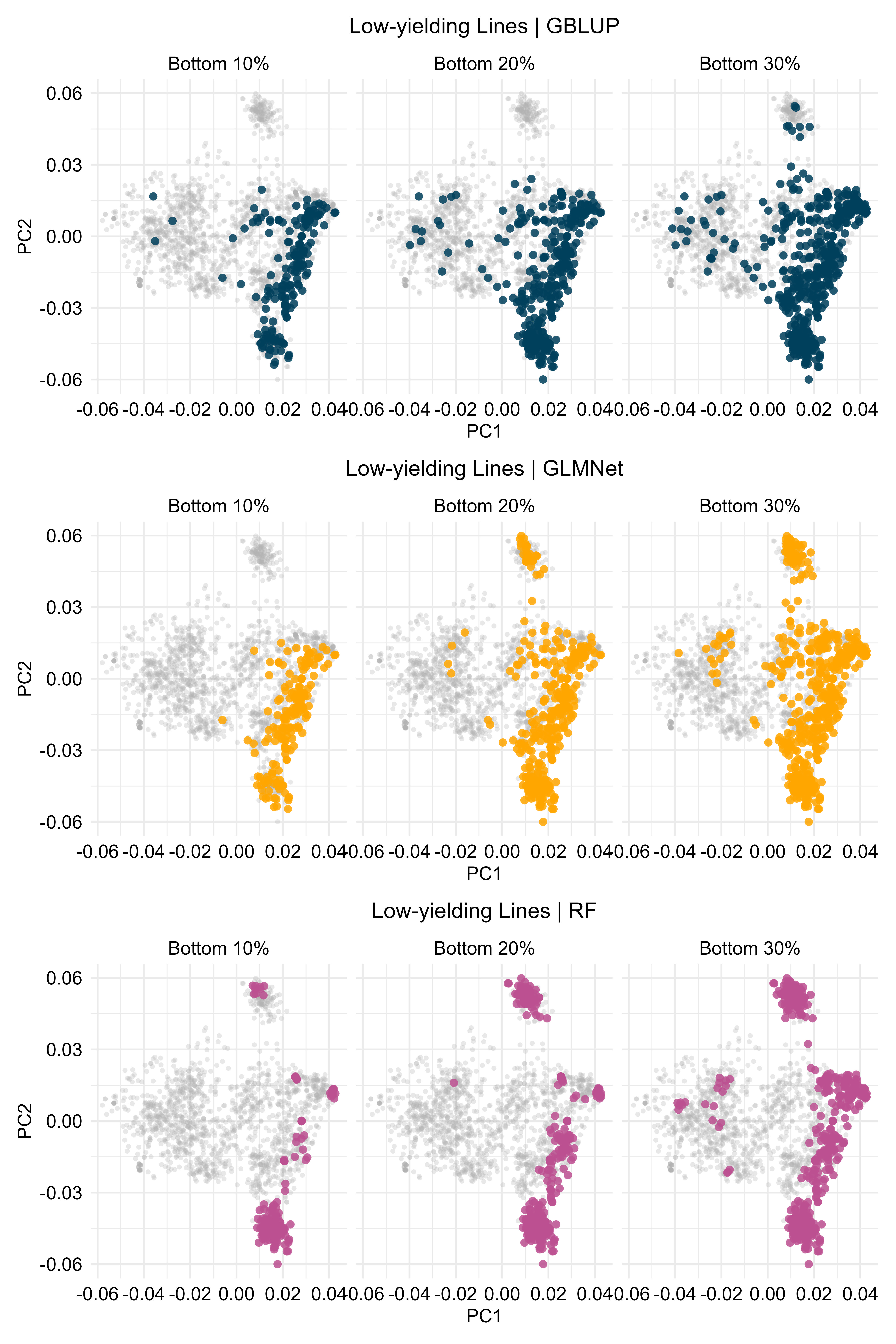

Supplement: Supplementary Figure 5 — Principal component analysis (PCA) of genomic marker data showing the genetic distribution of soybean genotypes selected as low-yielding according to averaged genomic prediction scores from three models (GBLUP, RF, and GLMNet). Colored points represent selected genotypes at three selection intensities (Top or Bottom 10%, 20%, and 30%), while gray points indicate the remaining unselected population. [file Image5.jpeg]

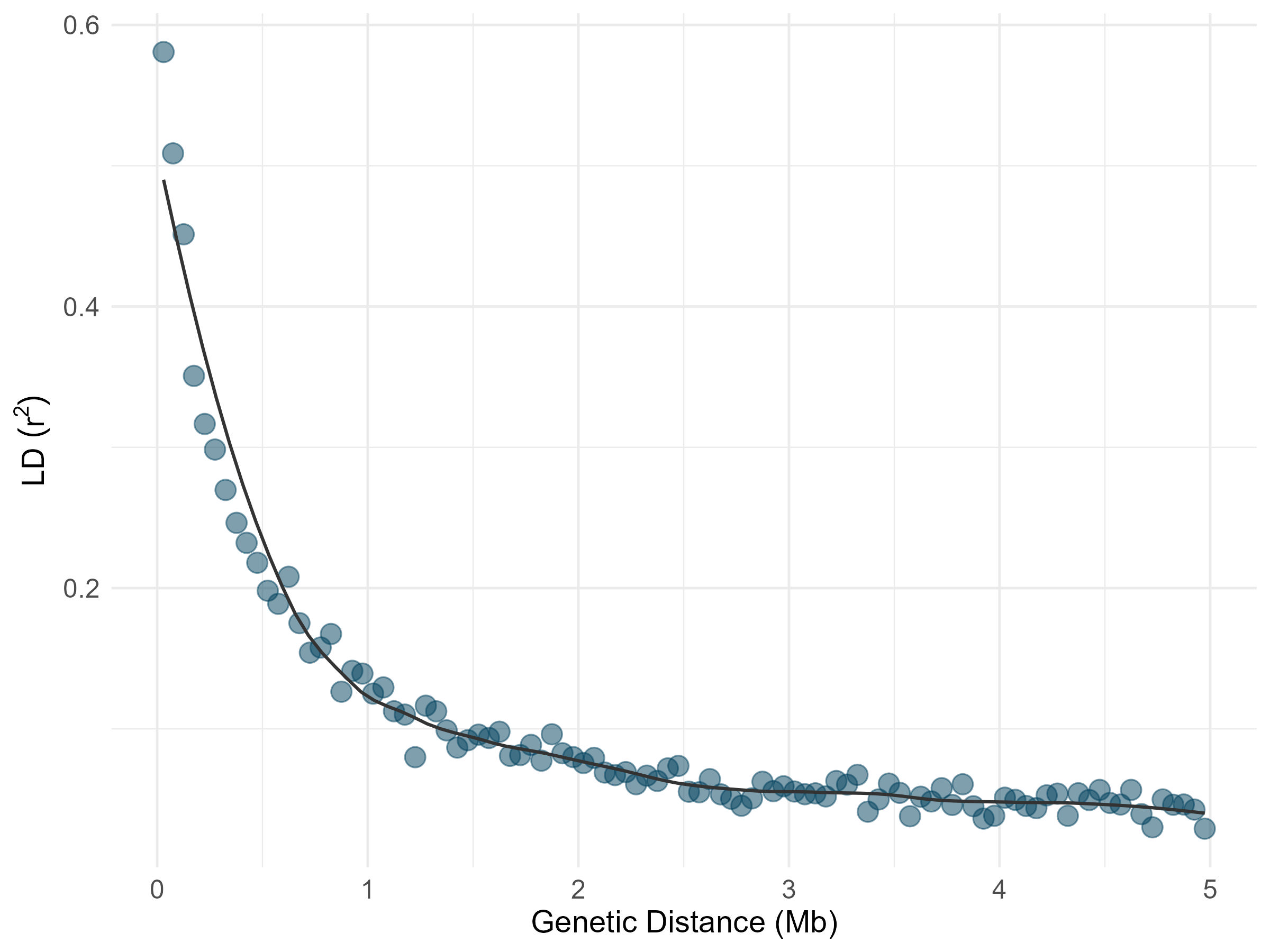

Supplement: Supplementary Figure 6 — Linkage disequilibrium (LD) decay estimated from genome-wide marker from breeding lines. Pairwise LD (r²) is plotted against physical genetic distance (Mb), with points representing average LD within distance bins and the solid line indicating the fitted decay trend. [file Image6.jpeg]
